# The effects of *Centella asiatica* (L.) Urban on neural differentiation of human mesenchymal stem cells in vitro

**DOI:** 10.1186/s12906-019-2581-x

**Published:** 2019-07-08

**Authors:** Norazzila Omar, Yogeswaran Lokanathan, Zainul Rashid Mohd Razi, Ruszymah Bt Haji Idrus

**Affiliations:** 1 Tissue Engineering Centre, Faculty of Medicine, Universiti Kebangsaan Malaysia Medical Centre, Jalan Yaacob Latif, Bandar Tun Razak, 56000 Cheras Kuala Lumpur, Malaysia; 2 Department of Obstetrics and Gynecology, Universiti Kebangsaan Malaysia Medical Centre, Jalan Yaacob Latif, Bandar Tun Razak, 56000 Cheras Kuala Lumpur, Malaysia; 3 Department of Physiology, Faculty of Medicine, Universiti Kebangsaan Malaysia Medical Centre, Jalan Yaacob Latif, Bandar Tun Razak, 56000 Cheras Kuala Lumpur, Malaysia

**Keywords:** *Pegaga*, Umbilical cord stem cells, Neural induction, Schwann cells, Neurotrophic factors

## Abstract

**Background:**

*Centella asiatica* (L.) Urban, known as Indian Pennywort, is a tropical medicinal plant from *Apiaceae* family native to Southeast Asian countries. It has been widely used as a nerve tonic in Ayuverdic medicine since ancient times. However, whether it can substitute for neurotrophic factors to induce human mesenchymal stem cell (hMSCs) differentiation into the neural lineage remains unknown. This study aimed to investigate the effect of a raw extract of *C. asiatica* (L.) (RECA) on the neural differentiation of hMSCs in vitro.

**Methods:**

The hMSCs derived from human Wharton’s jelly umbilical cord (hWJMSCs; *n* = 6) were treated with RECA at different concentrations; 400, 800, 1200, 1600, 2000 and 2400 μg/ml. The cytotoxicity of RECA was evaluated via the MTT (3-(4, 5-dimethylthiazolyl-2)-2, 5-diphenyltetrazolium bromide) and cell proliferation assays. The hWJMSCs were then induced to neural lineage for 9 days either with RECA alone or RECA in combination with neurotrophic factors (NF). Cell morphological changes were observed under an inverted microscope, while the expression of the neural markers S100β, p75 NGFR, MBP, GFAP and MOG was analyzed by quantitative polymerase chain reaction and immunocytochemistry. The cell cycle profile of differentiated and undifferentiated hWJMSCs was investigated through cell cycle analysis.

**Results:**

RECA exerted effects on both proliferation and neural differentiation of hWJMSCs in a dose-dependent manner. RECA reduced the proliferation of hWJMSCs and was cytotoxic to cells above 1600 μg/ml, with IC_50_ value, 1875 ± 55.67 μg/ml. In parallel with the reduction in cell viability, cell enlargement was also observed at the end of the induction. Cells treated with RECA alone had more obvious protein expression of the neural markers compared to the other groups. Meanwhile, gene expression of the aforementioned markers was detected at low levels across the experimental groups. The supplementation of hWJMSCs with RECA did not change the normal life cycle of the cells.

**Conclusions:**

Although RECA reduced the proliferation of hWJMSCs, a low dose of RECA (400 μg/ml), alone or in combination of neurotrophic factors (NF + RECA 400 μg/ml), has the potential to differentiate hWJMSCs into Schwann cells and other neural lineage cells.

**Electronic supplementary material:**

The online version of this article (10.1186/s12906-019-2581-x) contains supplementary material, which is available to authorized users.

## Background

Injuries to both central and peripheral nervous system, are increasing in number nowadays and have become a worrisome experience worldwide. In comparison with central nervous system, peripheral nervous system has a better self-recovery due to the response of Schwann cells that mediate the regeneration and remyelination of the axonal injuries. Nevertheless, number of Schwann cells still not up to par to recapitulate the healing process in cases of serious injuries.

The discovery of mesenchymal stem cells (MSCs) by Alexander Friedenstein half a century ago has brought new insights into nerve regeneration [1]. MSCs have been embraced as an attractive candidate for cell transplantation due to their ability to (1) secrete neurotrophic factors, (2) regulate inflammation through paracrine signaling and (3) activate resident stem cells to facilitate tissue repair [2, 3]. Several lines of evidence have successfully reported the transdifferentiation of MSCs derived from both tissues into Schwann-like cells using a combination of cytokines and recombinant/synthetic neurotropic factors [4–6]. However, rapid degradation and the high cost of growth factors have limited these applications for the treatment of neural regeneration, especially in developing countries [7, 8]. In addition, the recombinant/synthetic growth factors also have a greater tendency to stimulate the development of normal cells into cancer cells if used for a longer period [9]. Therefore, scientific investigations into neuropharmacological natural herbs have been urged in the search for new sources of nerve stimulants with minimal side effects, low toxicity and ready availability for the replacement of neurotrophic factors.

*Centella asiatica* (L.), which was used as a nerve tonic in Ayuverdic medicine since ancient times, has gained much attention from researchers to explore its medical benefits on the scientific basis. Generally, *C. asiatica* (L.) is a small perennial herbal plant with kidney-shaped leaves that belongs to the family *Apiceae* [10]. It grows in damp and swampy areas of tropical countries and is commonly known as *pegaga* in Malaysia, Indian Pennywort in the United States of America, *yuhong-yuhong* in the Philippines, *tapak kuda* in Indonesia and *buak bok* in Thailand [11, 12]. It has various therapeutic activities that are mainly attributed to its biologically active ingredients, i.e. triterpenes [13]. The triterpenes, which are comprised of asiatic acid, madecassic acid, asiaticoside and madecassoside, are used as biomarker components for *C. asiatica* (L.) [14]. In addition, *C. asiatica* (L.) is also rich of flavonoids, essential oils, amino acids, vitamins and minerals which may react synergistically with those bioactive compounds to elicit the therapeutic responses [15]. The bioactive components of *C. asiatica* (L.) have been demonstrated to have a maximum absorption in brain, skin and stomach and extensively distributed there and completely metabolized upon dosing [16].

Although excellent bioavailability of the crude extract of *C. asiatica* (L.) was seen in vitro, the bioavailability was lesser in vivo due to its poor lipid solubility and undesired molecular size [17]. Recently, *C. asiatica* (L.) extracts have been incorporated into nanoparticles to improve its solubility, absorption and stability for better in vivo drug delivery system [18]. Evidence has shown that asiatic acid derived-from *C. asiatica* (L.) can cross the blood-brain barrier (BBB) and the tight junction of BBB was maintained in the presence of *C. asiatica* (L.) extract [19, 20]. There was no any adverse effect of *C. asiatica* (L.) reported in vivo [21]. Nonetheless, side effects such as skin ulceration, extreme drowsiness, nausea and stomach ache potentially occur at the very high doses of this herbal plant [22].

The neuropharmacological value of *C. asiatica* (L.) has been widely investigated. It has been shown to have neuritogenic and neuroprotective effects on neural cells [23, 24]. However, most of these investigations assessed only the central nervous system. The effectiveness of *C. asiatica* on regeneration of the peripheral nervous system has not been elucidated yet [25]. Moreover, its biological activity in terms of promoting neural differentiation is poorly documented. Therefore, the present study aimed to investigate the effects of a raw extract of *C. asiatica* (L.) (RECA) on the differentiation of human Wharton’s jelly derived-mesenchymal stem cells (hWJMSCs) to Schwann cells in vitro.

## Methods

### Isolation and culture of hWJMSCs

The Universiti Kebangsaan Malaysia Research Ethics Committee approved the usage of human umbilical cord samples from consenting patients (UKM 1.5.3.5/244/FF-2015-217). Six samples of human umbilical cord (*n* = 6) were obtained with informed consent from women who had a healthy full-term pregnancy and who underwent a caesarian delivery. Isolation of hMSCs from human Wharton’s jelly of umbilical cord samples was performed using a method described by [26]. Briefly, human umbilical cord samples were washed thoroughly in sterile Dulbecco’s phosphate buffered saline (DPBS) (Sigma Aldrich, USA) to remove any blood. The cord tissues were dissected, and the cord vessels were removed to avoid endothelial cell contamination of the cell culture. The Wharton’s jelly part of the umbilical cord was minced and subjected to enzymatic digestion for 1.5 h in a shaking incubator at 37 °C. The digested tissue was then centrifuged at 5000 rpm for 5 min to obtain the hWJMSCs pellet. After washing, the cells were resuspended in low-glucose Dulbecco’s modified Eagle’s medium (DMEM-LG) (Gibco, USA) containing 10% fetal bovine serum (FBS) (Gibco, USA), 1% antibiotic-antimycotic (Gibco, USA), 2% HEPES buffer solution and 1% Glutamax (Gibco, USA). Then, the hWJMSCs were seeded at a density of 5 × 10^3^ cells/cm^2^ in 25 cm^2^ culture flask and incubated at 37 °C in humidified 5% CO_2_ incubator (Eppendorf, USA) with medium changes on alternate days. The cells were propagated until passage 4. The growth profile of the cultured cells, i.e. the percentage of total viable cells and the population doubling time, were recorded at every passage. Cultured cells were used at the passage with the greatest total cell viability and optimal proliferation rate in the subsequent analysis.

### Preparation of the raw extract of *C. asiatica* (L.) (RECA)

Fresh leaves of *C. asiatica* (L.) from Pulau Pinang, Malaysia were identified by Prof. Dr. Mohd Ilham Adenan from Atta-ur-Rahman Institute for Natural Product Discovery (auRIns), Universiti Teknologi MARA, Selangor, Malaysia and deposited at the institution (UiTM; voucher specimen no. CA-K017). RECA was prepared from powdered leaves of *C. asiatica* (L.) The leaves were washed, cleaned and dried in oven at 40 °C before being ground. A total of 50 kg of the powdered *C. asiatica* (L.) leaves was extracted in five batches. In each batch, 10 kg of *C. asiatica* (L.) leaves was extracted in 57% denatured ethanol (60 L of 95% ethanol + 40 L deionized water) for 8 h at 60 °C. A total of 14.8 L of concentrated liquid extract was produced following the extraction process. It was then freeze-dried to give a total of 7.96 kg of dried-powdered extract (15.92% yield). The powdered extract was recognized as raw extract of *C. asiatica* (L.) (RECA) and kept at room temperature until further use. The bioactive compounds of the extract were identified by High Performance Liquid Chromatography (HPLC) method.

### Cytotoxicity of RECA

RECA powder was dissolved directly in culture medium (DMEM-LG) and prepared at varying concentrations (400, 800, 1200, 1600, 2000 and 2400 μg/ml) before being used in the cell culture system. hWJMSCs at passage 3 (P3) were cultured triplicates in 48-well plates at a density of 5 × 10^3^ cells/cm^2^ in DMEM-LG containing 10% fetal bovine serum (FBS) for 24 h. Then, the medium was discarded, and the cells were supplemented with different concentrations of RECA in DMEM-LG for another 24 h at 37 °C in a 5% CO_2_ incubator. The hWJMSCs without RECA supplementation served as the control. At the end of the assay, the morphology of the cells was recorded, and cell viability was measured using the Vibrant® MTT Cell Proliferation Assay Kit (Invitrogen, USA). The assay was performed according to the protocol provided by the manufacturer. Briefly, 10 μl of MTT stock solution (12 mM) with 100 μl of fresh medium was pipetted into the wells and the plate was incubated for 4 h at 37 °C in the dark. 100 μl SDS-HCL solution was then added to the wells and incubated again for another 4 h. The absorbance in each well was measured at 570 nm using a spectrophotometer (Bio-TEK, USA). The cell viability at each RECA concentration was expressed as a percentage relative to the untreated cells (control). The IC_50_ value of RECA (inhibitory concentration causing a 50% reduction in the cell population) was calculated from the dose-response curve to determine the exposure limit of RECA in hWJMSCs.

### Proliferation assay with RECA

In conjunction with the cytotoxicity assay, the proliferation rate of hWJMSCs after RECA treatment was also assessed to investigate the long-term tolerance of hWJMSCs to RECA. Three independent samples of hWJMSCs (P3) at a density of 3 × 10^3^ cells/cm^2^ were cultured in 12-well plates for 24 h before RECA supplementation at varying concentrations (400, 800, 1200, 1600 and 2000 μg/ml). The cells were further incubated in a 5% CO_2_ incubator at 37 °C for 168 h. hWJMSCs, which served as the control, were cultured without RECA in DMEM-LG supplemented with 10% FBS. The medium was changed every 48 h. The distribution of the cells in culture was recorded at different time points (24 h, 72 h, 120 h and 168 h after RECA treatment) using an inverted microscope (Nikon A1_R_, Japan). The number of viable cells for each RECA concentration was calculated based on image analysis. The proliferation rate of the cells in each treatment group was analyzed and presented as the cell concentration using the following formula:

$$ {\displaystyle \begin{array}{l} Cell\ concentration\ \left( cells/\upmu {m}^2\right)=a/b\\ {}a: total\ number\ of\ counted\ cells\ in\ an\ image\ (cells)\\ {}b: area\ of\ the\ captured\ image\ \left(\upmu {m}^2\right)\end{array}} $$

The assay was also conducted on human Schwann cells to investigate the tolerance of neural cells for RECA by determining the suitable concentrations of RECA for use in the neural differentiation of hWJMSCs. Human Schwann cells were purchased from ScienCell Research Laboratories (SanDiego, USA). The cells were treated with RECA as previously described, and the cell viability in each experimental group was determined using the MTT assay.

### In vitro differentiation of hWJMSCs into the Schwann cell lineage

The neural differentiation of hWJMSCs was performed for 9 days as previously described [4, 27] with minor modifications. hWJMSCs at passage 3 were grouped into four groups. Group 1 (undifferentiated) served as the negative control, where hWJMSCs at a density of 3 × 10^3^ cells/cm^2^ were maintained in alpha-Minimum Essential Medium (α-MEM) supplemented with 10% FBS, 1% antibiotic-antimycotic, 2% HEPES buffer solution and 1% Glutamax throughout the induction. Cells in Group 2 (neurotrophic factors; NF) underwent three phases of induction. Initially, hWJMSCs were cultured for 24 h in α-MEM for attachment. After that, the medium was changed, and the cultured cells were incubated with pre-induction medium containing 1 mM β-mercaptoethanol (BME) (Gibco, USA) without serum for 24 h. Then, the medium was replaced with α-MEM containing 10% FBS and 35 ng/ml all-trans retinoic acid (ATRA) (Sigma Aldrich, USA) after 3 days of culture. Next, the cells were induced into Schwann cells using α-MEM containing 10% FBS and a mix of neurotropic factors for another 5 days. The neurotrophic factor mix consisted of 5 μM forskolin, 10 ng/ml recombinant human basic fibroblast growth factor (bFGF), 5 ng/ml human recombinant platelet-derived growth factor-AA (PDGF-AA) and 200 ng/ml human recombinant NRG1-beta 1/HRG1-beta 1 EGF-domain, which were purchased from R&D Systems, Inc. (USA). hWJMSCs in Group 3 underwent a similar induction process as the cells in Group 2, except that induction medium, i.e. α-MEM containing 10% FBS and neurotrophic factors, was also supplemented with different concentrations of RECA (400, 1200 and 2000 μg/ml). In contrast to the other induction groups, hWJMSCs in Group 4 were induced with RECA alone (400, 1200 and 2000 μg/ml) in α-MEM throughout the culture. Human Schwann cells were cultured along this assay as a positive control.

### Gene expression analysis of differentiated hWJMSCs

Total RNA was isolated using TRIzol® reagent (Life Technology, USA) and the isolated RNA was used to synthesize cDNA using the QuantiNova™ Reverse Transcription kit according to the manufacturer’s instructions (Qiagen, Germany). QuantiNova® SYBR® Green PCR (Qiagen, Germany) was used for quantitative polymerase chain reaction (qPCR) analysis. The assay was run in a StepOne Plus Real-Time thermal cycler (Applied Biosystems, USA) with the following PCR cycling conditions: initial denaturation for 2 min at 95 °C, followed by 40 cycles of denaturation for 5 s at 95 °C, then annealing/extension for 10 min at 60 °C for all the primer pairs except for p75 NGFR at 64 °C. The assay was followed by a melt curve analysis to ensure PCR product specificity. The primer sequences and the amplicon sizes are listed in Table 1. The gene expression level of neural markers was normalized to the reference gene glyceraldehyde 3-phosphate dehydrogenase (GAPDH).

**Table 1:** The primer pairs used in the qPCR assay and amplicon sizes

Gene	Primer Sequences	Product size (bp)
GAPDH	Forward 5′- AGCCTCAAGATCATCAGCAATGCC-3′ Reverse 5′- TGGACTGTGGTCATGAGTCCTTCC-3’	110
S100β	Forward 5’- GGAAGGGGTGAGACAAGGA-3′ Reverse 5′- GGTGGAAAACGTCGATGAG-3’	73
p75 NGFR	Forward 5’- AACAAGACCTCATAGCCAGCACGG −3′ Reverse 5′- AGCTGTTCCACCTCTTGAAGGC-3’	174
MBP	Forward 5’-CTTCAAGAACATTGTGACGCCTCG-3′ Reverse 5′- GTGAGCCGATTTATAGTCGGACG-3’	148
GFAP	Forward 5’-ACCTGCAGATTCGAGAAACCAGC-3′ Reverse 5′-ACATCCTTGTGCTCCTGCTTGG-3’	136

*GAPDH* Glyceraldehyde 3-phosphate dehydrogenase, *p75 NGFR* Low-affinity nerve growth factor receptor, *MBP* Myelin binding protein, *GFAP* Glial fibrillary acidic protein, *bp* Base pair

### Immunocytochemical analysis of differentiated hWJMSCs

Cells were fixed with 4% paraformaldehyde, blocked with normal goat serum and incubated with primary antibodies. The primary antibodies used were a mouse monoclonal antibody against S100β (1:1000, Abcam), a rabbit polyclonal antibody against low affinity nerve growth factor receptor (p75 NGFR) (1:1000, Abcam), a mouse monoclonal antibody against myelin binding protein (MBP) (1:1000, Thermo Fisher), a mouse monoclonal antibody against glial fibrillary acidic protein (GFAP) (2 μg/ml, Stem Cell™ Technologies) and a mouse monoclonal antibody against myelin oligodendrocyte glycoprotein (MOG) (2 μg/ml, Abcam). Alexa Fluor® 488 goat anti-rabbit IgG (1:300, Abcam) and Texas Red® goat anti-mouse IgG (1:300, Abcam) were used as the secondary antibodies. An internal control for the assay was included by omitting the primary antibodies from the samples. Cell nuclei were counterstained with 4′, 6-diamidino-2-phenylindole (DAPI). The cells in various groups were examined by fluorescence microscopy (Nikon A1_R_, Japan) using the same laser intensity and detection sensitivity. The expression of all tested neural markers in differentiated and undifferentiated hWJMSCs was analyzed qualitatively and compared to the positive control.

### Cell cycle analysis of hWJMSCs

The cell cycle phase distribution of differentiated and undifferentiated hWJMSCs was analyzed using the BD Cycletest™ Plus DNA kit (Becton Dickinson, USA) as recommended by the manufacturer. Cells were harvested by trypsinization, centrifuged and resuspended in 1 ml of buffer solution. A total of 1 × 10^6^ cells/ml was used for this assay. The cells were stained with propidium iodide (PI) and then incubated at 4 °C for 10 min in the dark. The assay was conducted using a BD FACSVerse™ flow cytometer (Becton Dickinson, USA) on three independent samples. The raw data were collected using CELLQuest software (Becton Dickinson, USA), while the cell cycle profile was analyzed using ModFit LT™ version 4.1 (Verity House Software, Topsham, USA).

### Statistical analysis

Data are represented as mean ± standard error mean (SEM). The data from each experimental group were analyzed using GraphPad Prism version 7.0 (GraphPad Software, Inc., USA) and compared using one-way ANOVA. A *p* < 0.05 was considered statistically significant.

## Results

### Phenotypic features of hWJMSCs

**Fig. 1:**
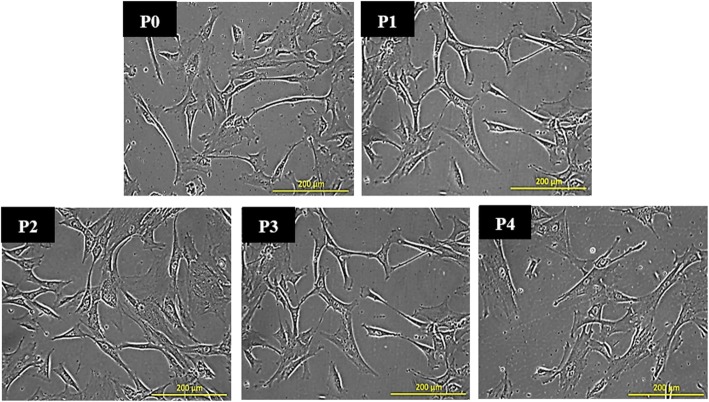
Phase contrast images of hWJMSCs in culture from the initial passage (P0) to passage 4 (P4). The cells were small to large and exhibited a flat and bipolar/multipolar fibroblastic morphology throughout the culture (*n* = 6). Scale bar 200 μm

Observation by phase contrast microscopy showed that hWJMSCs were small to large and exhibited the flat and bipolar/multipolar fibroblastic morphological features of MSCs. This fibroblastic appearance was retained from the initial passage (P0) to the late passage (P4), as depicted in Fig. 1. The cultured cells were characterized and confirmed as MSCs in a previous study performed in our laboratory based on the minimal criteria for defining MSCs, where the isolation of MSCs from umbilical cord was conducted using the same reagents and methods as those used in the present study [26].

**Fig. 2:**
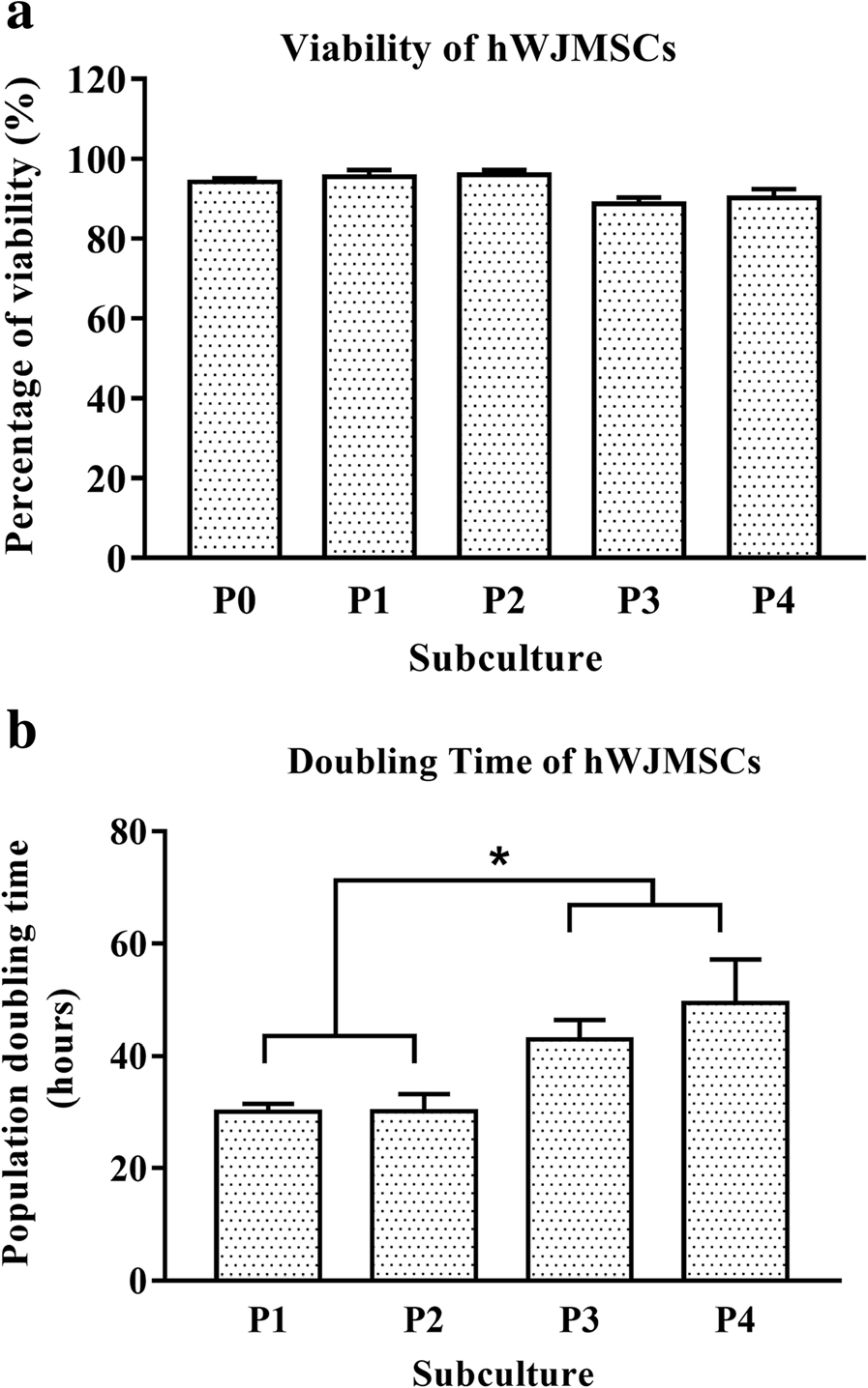
Growth kinetic profile of hWJMSCs. **a** Percentage of viability of hWJMSCs. **b** Population doubling time of hWJMSCs from the initial passage (P0) until passage 4 (P4). * indicates a significant difference at *p* < 0.05 (*n* = 3)

Based on the growth kinetic analysis, hWJMSCs exhibited more than 80% viability at all passages (Fig. 2). Nonetheless, the ability of hWJMSCs to proliferate in culture became slower at higher passage numbers. Figure 2 demonstrates that the proliferation of hWJMSCs was significantly reduced after cells were subcultured beyond passage 2 (P2), *p* < 0.05. The hWJMSCs at passage 3 and 4 required a longer time to proliferate (42.88 ± 3.52 h and 49.39 ± 7.76 h respectively) compared to hWJMSCs at passage 1 and 2. Nevertheless, hWJMSCs at passage 3 were chosen due to the insufficient number of hWJMSCs at the earlier passages (P1 and P2).

### Phytochemical profile of RECA

The result of HPLC analysis demonstrated that RECA contained all four bioactive components of *C. asiatica* (L.) as shown in Additional file 1: Figure S1. Those compounds are madecassoside (0.0060%), asiaticoside (0.0035%), madecassic acid (0.0020%) and asiatic acid (0.0017%). Moreover, RECA also contained 206.73 ± 5.53 mg/100 g gallic acid equivalent (GAE) of total phenolic content and was found to have 57.70 ± 0.78% antioxidant activity via DPPH (2,2-diphenyl-1-picrylhydrazyl) radical scavenging assay.

### Cytotoxic effects of RECA on hWJMSCs

**Fig. 3:**
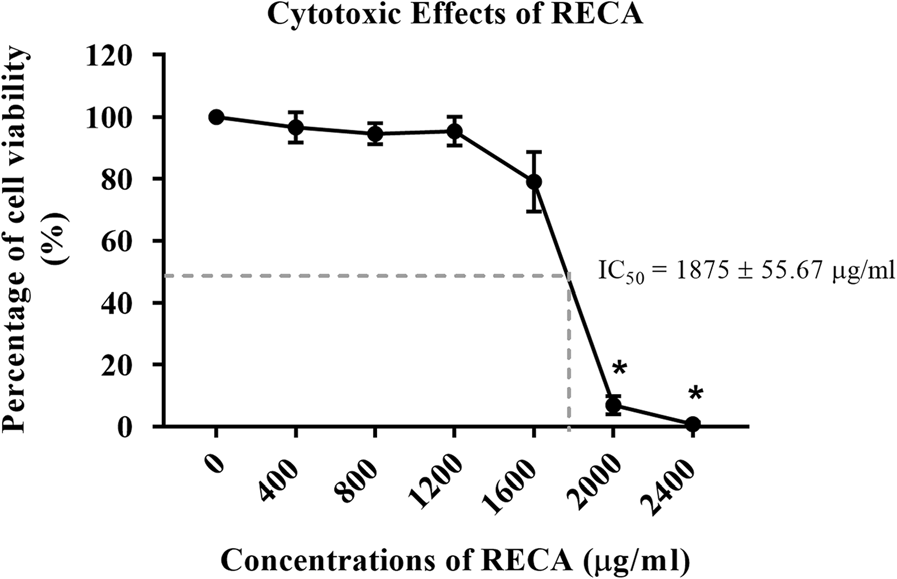
Dose-dependent effects of RECA on hWJMSCs. The viability of hWJMSCs was reduced after supplementation with RECA for 24 h. RECA at 2000 μg/ml and 2400 μg/ml significantly inhibited the growth of hWJMSCs, *p* < 0.05 (*n* = 6)

The study on the effects of RECA on neural differentiation of hWJMSCs was preceded by assessing the cytotoxicity of the extract on the cells. The analysis showed that the supplementation of hWJMSCs with RECA reduced cell growth in a dose-dependent manner. As shown in Fig. 3, more inhibitory effects on cell growth were observed as the concentration of RECA increased. RECA at higher concentrations (2000 and 2400 μg/ml) significantly inhibited the growth of hWJMSCs, which reduced cell viability at these RECA concentrations to 6.96 ± 2.92% and 0.74 ± 0.67%, respectively (*p* < 0.05). The findings demonstrate that RECA above 1200 μg/ml is cytotoxic to hWJMSCs after 24 h of culture. The determined IC_50_ value was 1875 ± 55.67 μg/ml.

**Fig. 4:**
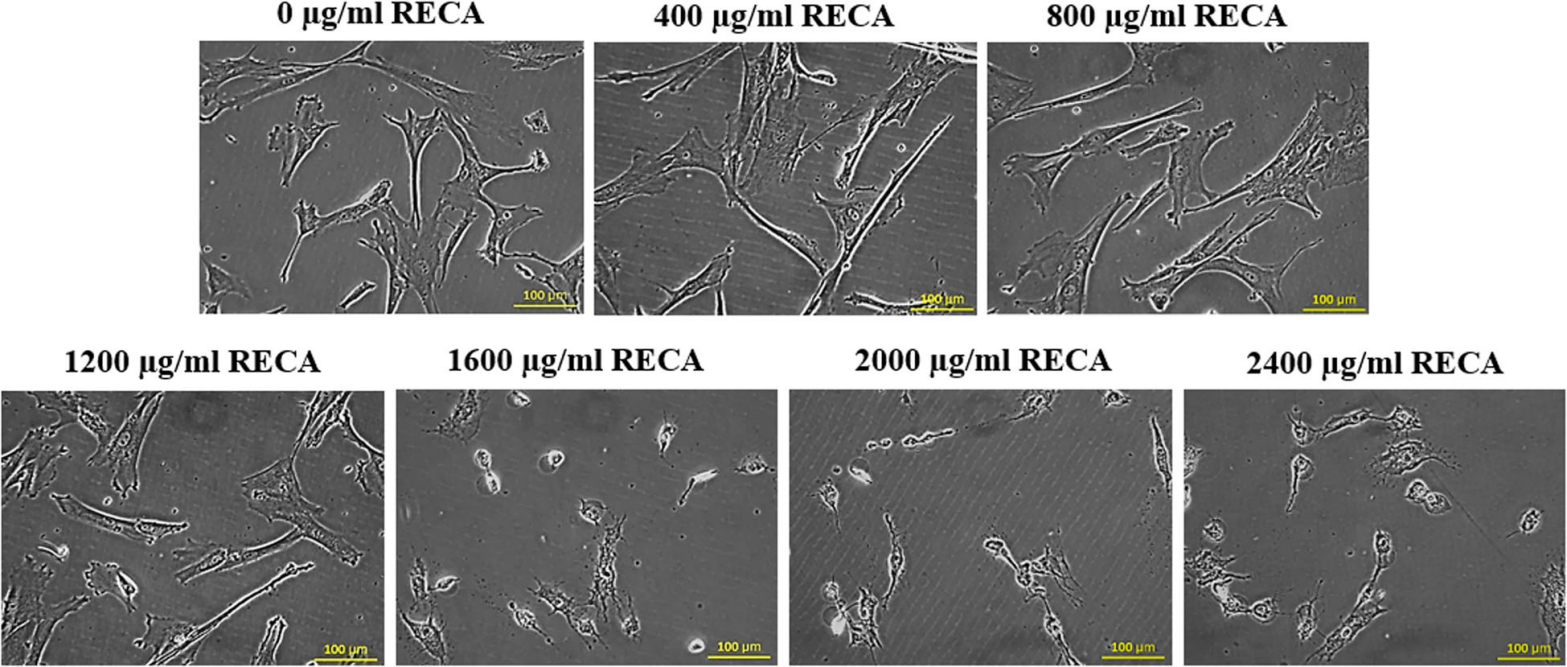
Morphological changes in hWJMSCs after 24 h of treatment with varying concentrations of RECA. The hWJMSCs underwent cell damage with the supplementation of RECA above 1200 μg/ml (*n* = 6). Scale bar 100 μm

In parallel to cell viability, hWJMSCs also underwent morphological changes after supplemented with varying concentrations of RECA. As shown in Fig. 4, the morphological changes to hWJMSCs were noted after 24 h of culture with RECA, particularly at 1200 μg/ml. This phenomenon was more obvious as the concentrations of RECA increased up to 1600 μg/ml until 2400 μg/ml, after which point the treated hWJMSCs had lost membrane integrity and were lysed at the end of culture (Fig. 4).

**Fig. 5:**
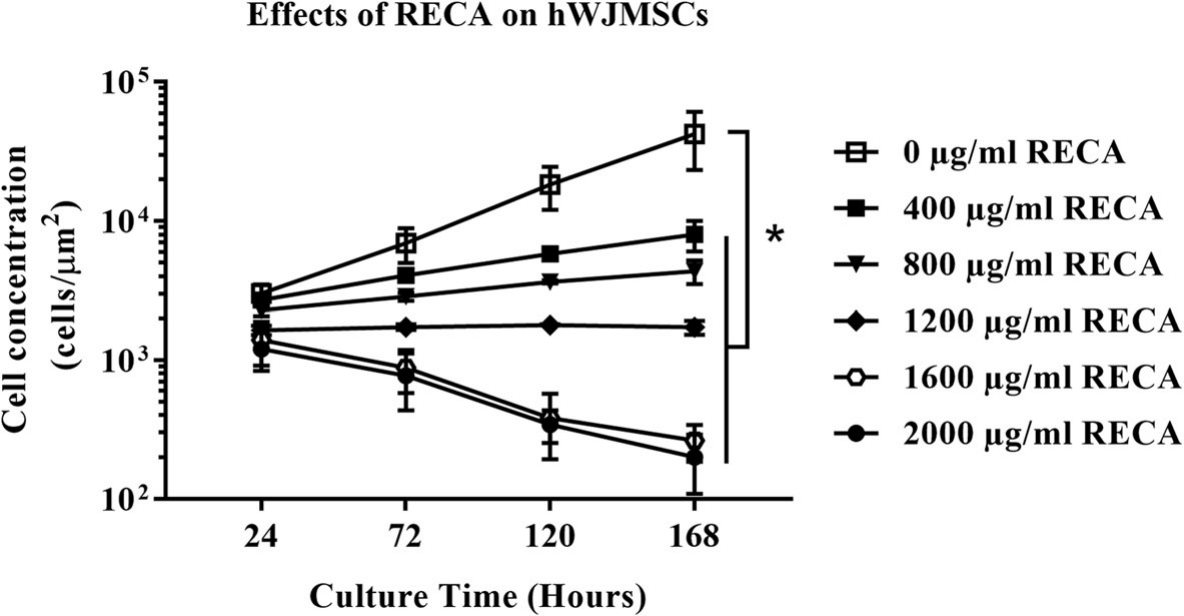
The anti-proliferative of RECA on hWJMSCs. The hWJMSCs were treated with varying concentrations of RECA for 168 h and the cell proliferation was assessed at different time intervals, which is after 24, 72, 120 and 168 h of RECA treatment. The proliferation rate of hWJMSCs was significantly reduced after 168 h of treatment with various concentrations of RECA compared to untreated hWJMSCs, which proliferated throughout the culture period, *p < 0.05 (*n* = 3)

The cytotoxic effects of RECA were further analyzed by proliferation assays in order to observe the long-term effects on hWJMSCs. As shown in Fig. 5, the proliferation rate of hWJMSCs was significantly reduced after supplementation with varying concentrations of RECA for 168 h (*p* < 0.05). Although RECA at 400, 800 and 1200 μg/ml was not found to be cytotoxic to hWJMSCs, it was, however, found to have anti-proliferative effect on these cells. RECA, particularly at 400 μg/ml, significantly reduced the concentration of hWJMSCs to 8027 ± 1985.75 cells/μm^2^ compared to the control (untreated hWJMSCs). The concentration of hWJMSCs was further decreased to 4354.27 ± 841.67 cells/μm^2^ and 1727.86 ± 199.66 cells/μm^2^ after treatment with RECA at 800 μg/ml and 1200 μg/ml, respectively. RECA above a dose of 1600 μg/ml inhibited the proliferation of hWJMSCs; the cell concentrations for RECA at 1600 μg/ml and 2000 μg/ml were 264.10 ± 78.21 cells/cm^2^ and 199.68 ± 90.82 cells/μm^2^, respectively.

**Fig. 6:**
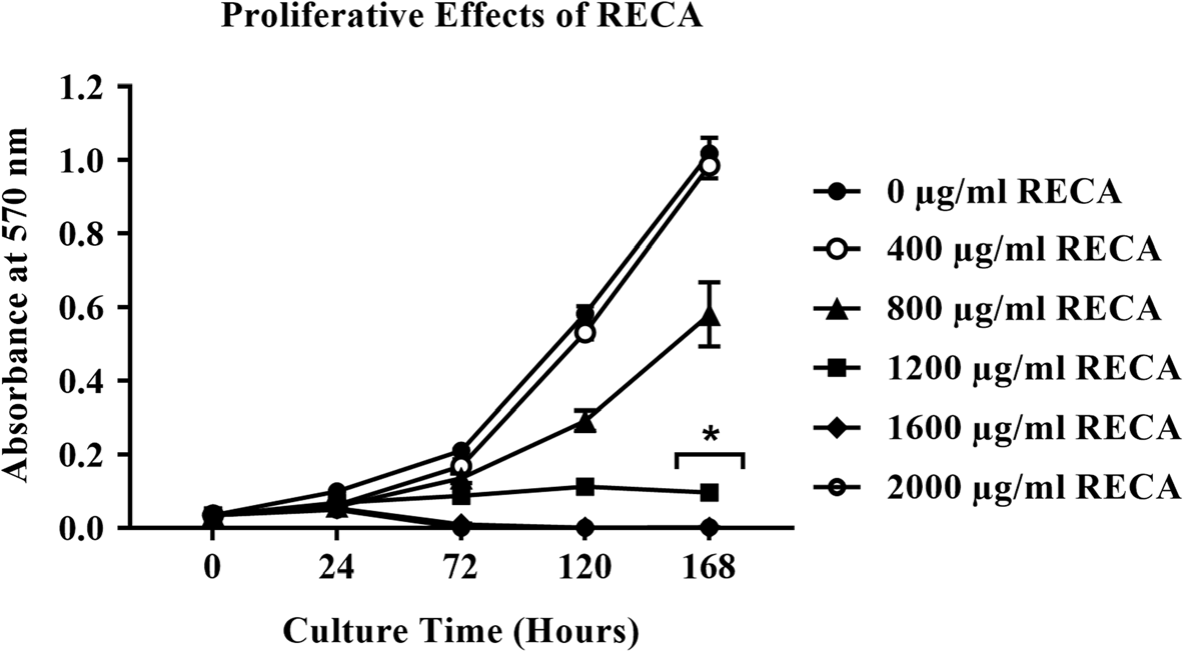
The effects of RECA on the proliferation of Schwann cells. The cell viability for each concentration of RECA was analyzed using the MTT assay at different time points (24 h, 72 h, 120 h and 168 h of RECA treatment). The absorbance was read at 570 nm and the value represents the mean ± SEM (*n* = 3). * indicates significant differences between the viability of Schwann cells treated with RECA at ≥1200 μg/ml compared to the viability of control (untreated Schwann cells) after 168 h in culture, *p* < 0.05

In parallel to hWJMSCs, Schwann cells also experienced reduced proliferation after supplementation with RECA in a similar concentration range. Nonetheless, a distinct inhibitory trend on cell proliferation was only noted after 72 h of RECA treatment, as depicted in Fig. 6. In contrast to the other experimental groups, Schwann cells treated with RECA at 400 μg/ml exhibited a similar pattern of proliferation as the control (untreated Schwann cells) throughout 168 h of culture. RECA at 800 μg/ml was found to reduce the proliferation of Schwann cells by the end of the assay. Nevertheless, the reduction in the proliferation rate of this particular group of cells was insignificant compared to the control (untreated Schwann cells), *p* < 0.05. The proliferation rate of Schwann cells was found to be significantly inhibited after supplementation with RECA at 1200 μg/ml and above for 168 h, *p* < 0.05.

The cytotoxicity analysis demonstrated that hWJMSCs and Schwann cells exhibited different degrees of tolerance to RECA. However, RECA at higher concentrations (≥1200 μg/ml) was toxic to both types of cells. Therefore, RECA concentrations at below 800 μg/ml were chosen for the neural differentiation of hWJMSCs. The present study also included supplementation of RECA at a final concentration of 1200 μg/ml and 2000 μg/ml in the neural differentiation assay to examine its neural stimulatory effects on hWJMSCs at higher concentrations.

### Effects of RECA on the neural differentiation of hWJMSCs into the Schwann cell lineage

#### Morphological changes to hWJMSCs

**Fig. 7:**
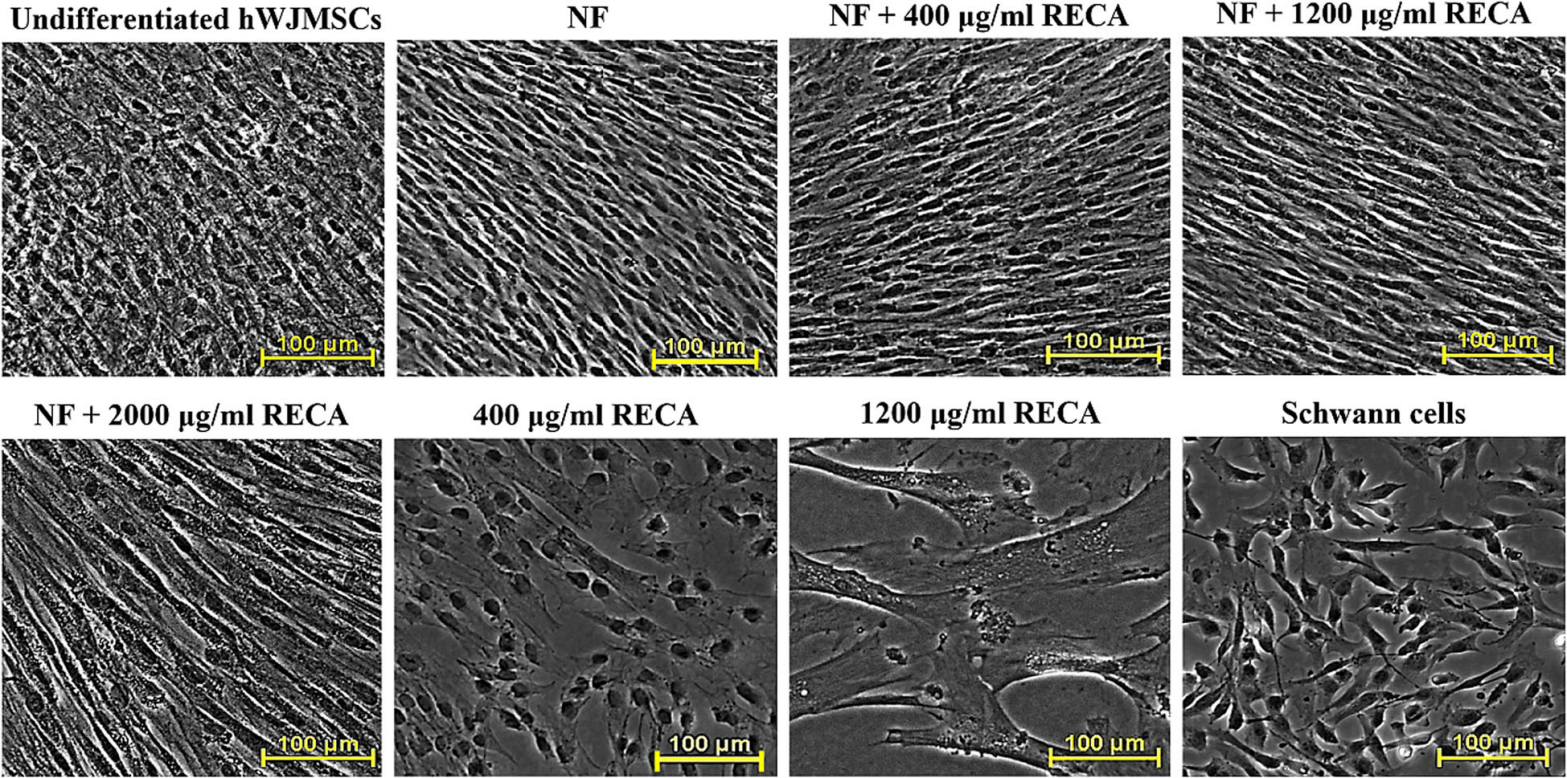
Morphological changes to hWJMSCs after 9 days of neural induction in different treatments. The undifferentiated hWJMSCs served as negative control, with Schwann cells as the positive control. Differentiated hWJMSCs in NF and all NF + RECA groups displayed a spindle-like cell morphology. A reduction in cell number was detected in the presence of RECA and cell enlargement was clearly noted with an increase in the RECA concentration (*n* = 6). NF; neurotrophic factors. Scale bar 100 μm

Undifferentiated hWJMSCs were very confluent at the end of the induction period (Fig. 7), and this caused difficulties with the morphological analysis by phase-contrast microscopy. A similar situation was also noted on the differentiated hWJMSCs in the NF group. However, the cells in this group exhibited a spindle-like morphology that was similar to Schwann cells (positive control). This morphological appearance was also observed in differentiated hWJMSCs in all NF + RECA groups. However, RECA treatment reduced cell numbers, whilst the differentiated hWJMSCs became enlarged with an increase in the RECA concentration. Cell enlargement was clearly observed whenever the hWJMSCs were induced with RECA alone, particularly at 400 μg/ml and 1200 μg/ml (Fig. 7). The hWJMSCs that were induced with 2000 μg/ml RECA were dead at the end of the induction period (data not shown).

#### Gene expression of neural-specific markers

**Fig. 8:**
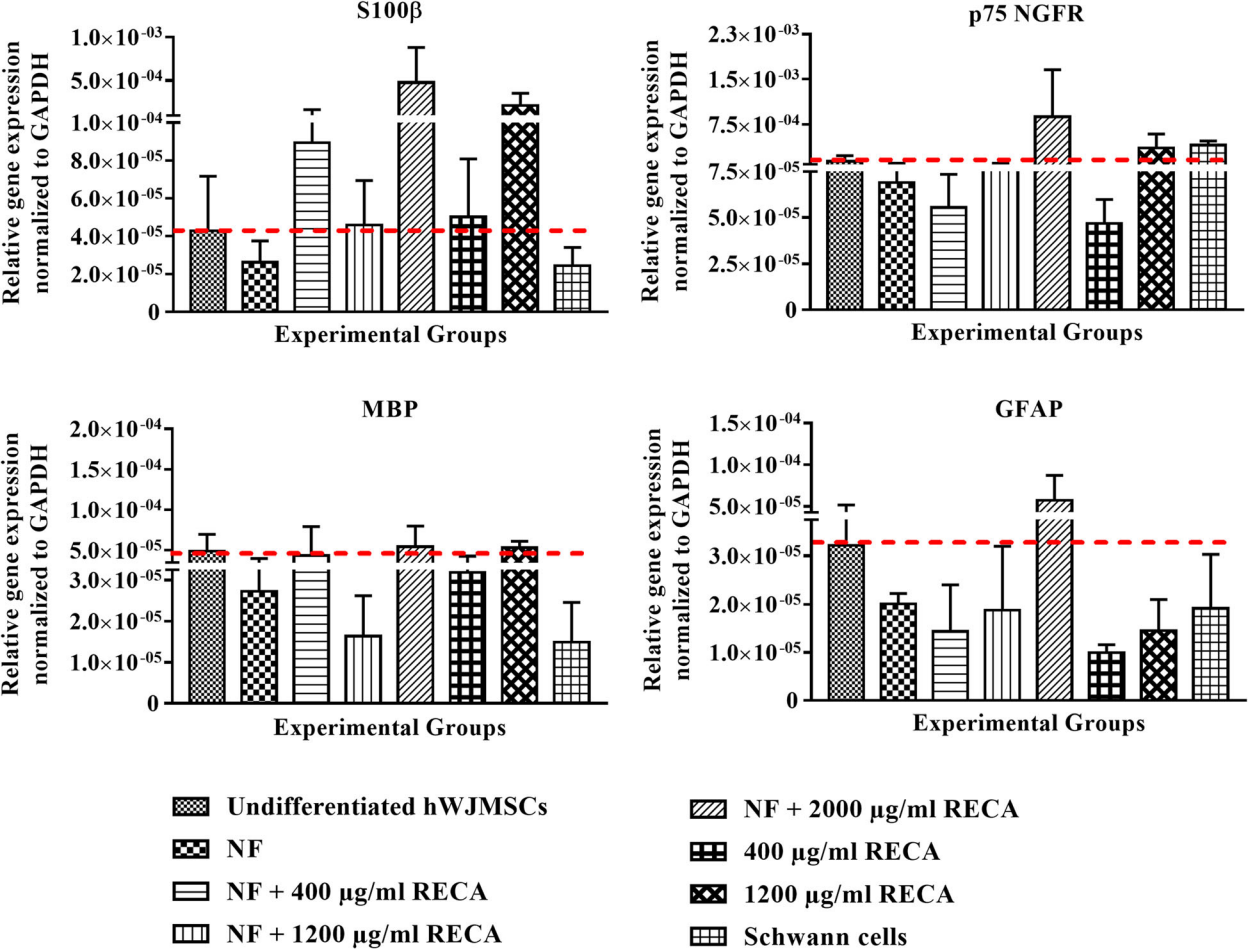
Gene expression analysis of S100β, p75 NGFR, MBP and GFAP in differentiated hWJMSCs from different induction groups. Data are presented as relative gene expression normalized to the housekeeping gene, GAPDH. The gene expression level of all the neural gene markers was not significantly different among experimental groups, *p <* 0.05 (*n* = 3). GAPDH; glyceraldehyde 3-phosphate dehydrogenase

Gene expression analysis was performed to detect the expression level of Schwann cell- specific markers in differentiated hWJMSCs. The findings show that the gene expression level of all of the tested neural markers was very low regardless of the induction group (Fig. 8). Nevertheless, hWJMSCs induced with NF + 2000 μg/ml RECA appeared to have the highest expression level of S100β among of the other groups. The expression of this gene marker was upregulated 7-fold in this particular group compared to its expression in undifferentiated hWJMSCs. However, no significant differences were detected between groups (*p* < 0.05).

In parallel to S100β, the gene expression of p75 NGFR was upregulated 5-fold (8.78 × 10^− 4^ ± 7.81 × 10^− 4^) in the NF + 2000 μg/ml RECA group compared to the undifferentiated group (1.34 × 10^− 4^ ± 9.80 × 10^− 5^), but the difference was insignificant (*p* < 0.05). Amongst the other experimental groups, hWJMSCs differentiated with 400 μg/ml RECA alone had the lowest expression level of the neural marker p75 NGFR (4.68 × 10^− 5^ ± 1.30 × 10^− 5^).

The differentiated hWJMSCs in the NF + 400 μg/ml, NF + 2000 μg/ml and 1200 μg/ml RECA groups had a similar expression level of the neural gene MBP as the undifferentiated hWJMSCs (Fig. 8). The transcription level of this Schwann cell marker was reduced in differentiated hWJMSCs in the NF + 1200 μg/ml RECA group. A similar expression profile was also reported in Schwann cells, used as the positive control in the analysis.

In addition to S100β and p75 NGFR, differentiated hWJMSCs in the NF + 2000 μg/ml RECA group showed the highest expression level of GFAP (5.72 × 10^− 5^ ± 2.30 × 10^− 5^). The lowest transcription level of GFAP was detected in differentiated hWJMSCs in the 400 μg/ml RECA alone group (9.90 × 10^− 6^ ± 1.70 × 10^− 6^), which was 3-fold lower than in undifferentiated hWJMSCs (3.22 × 10^− 5^ ± 1.95 × 10^− 5^). However, the difference was insignificant (*p* < 0.05).

#### Protein expression of neural-specific markers in differentiated hWJMSCs

**Fig. 9:**
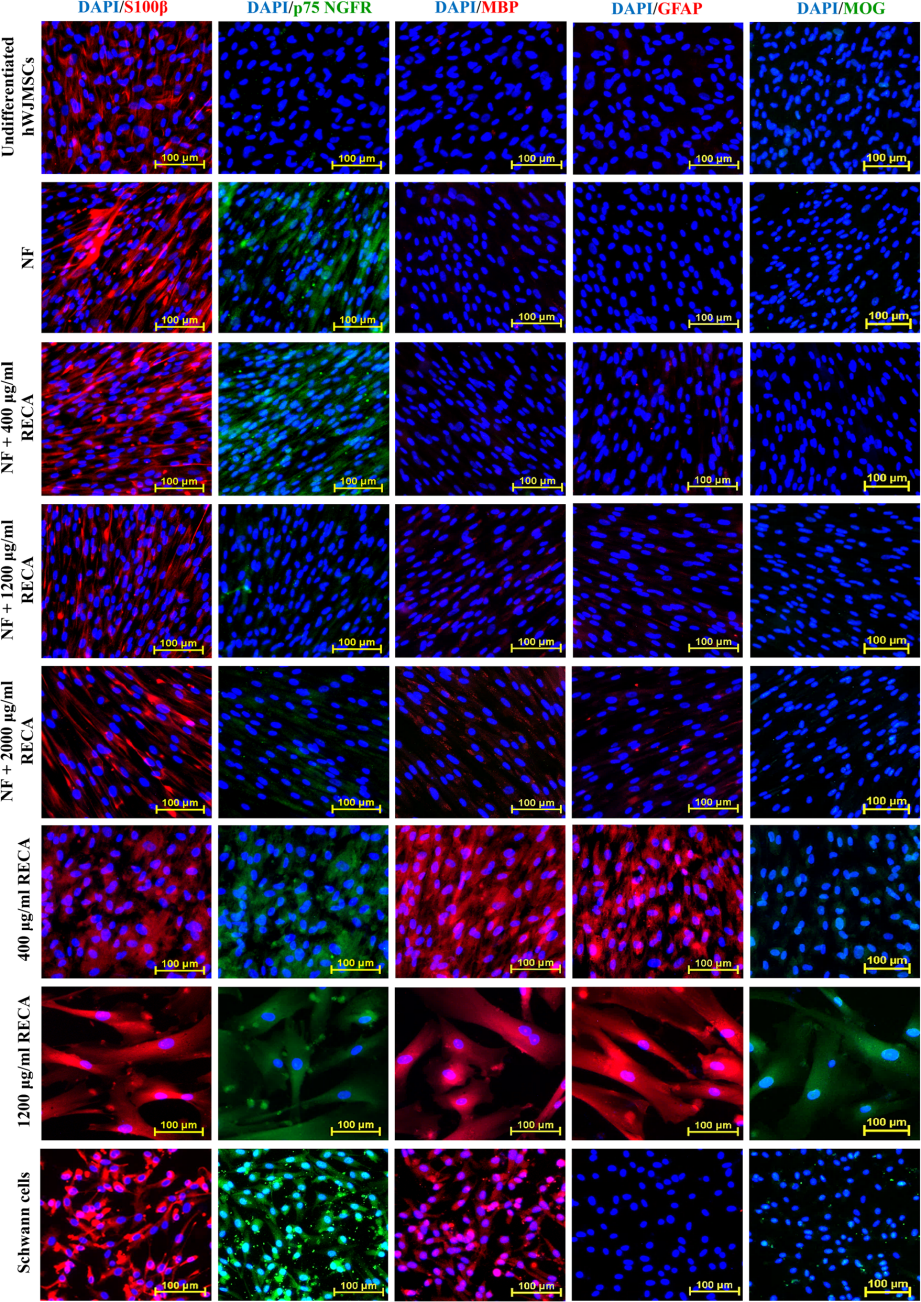
Immunocytochemical analysis of S100β, MBP, GFAP (red) as well as p75 NGFR and MOG (green) in differentiated and undifferentiated hWJMSCs. Cell nuclei were counterstained with DAPI (blue). Undifferentiated hWJMSCs served as a negative control, while Schwann cells acted as a positive control. hWJMSCs differentiated with 1200 μg/ml RECA showed prominent expression of all neural-specific markers compared to differentiated hWJMSCs in other induction groups (*n* = 6). DAPI; 4′, 6-diamidino-2-phenylindole. Scale bar 100 μm

Immunocytochemical analysis revealed that the hWJMSCs in all differentiated groups had higher expression of S100β, a Schwann cell-related marker (Fig. 9). The expression of this protein was also observed in undifferentiated hWJMSCs. Nevertheless, the latter did not show the expression of other Schwann cell markers, i.e. p75 NGFR and GFAP. The expression of p75 NGFR protein was detected at a low level in NF + 1200 μg/ml RECA and NF + 2000 μg/ml RECA-induced hWJMSCs. High expression of p75 NGFR protein was detected in both the NF and NF + 400 μg/ml RECA induction groups. Nevertheless, the expression level of this neural protein marker was inversely proportional to its transcription level in the 400 μg/ml RECA induction group. A similar expression profile was also reported in Schwann cells, as well as hWJMSCs that were induced using RECA alone at 1200 μg/ml (Fig. 9).

The expression of MBP protein was detected at a low level in both NF + 1200 μg/ml and NF + 2000 μg/ml RECA-induced hWJMSCs. A similar expression profile was also reported in differentiated hWJMSCs in the NF and NF + 400 μg/ml RECA induction groups. High expression of MBP was only observed when hWJMSCs were induced with RECA alone (400 μg/ml and 1200 μg/ml) (Fig. 9).

In addition to Schwann cell markers, markers of other neural cell types, i.e. GFAP and MOG were also used to investigate whether RECA has the potential to differentiate hWJMSCs into other neural cell lineages. The immunocytochemical analysis reported that hWJMSCs induced with RECA alone (400 μg/ml and 1200 μg/ml) had higher protein expression of GFAP than hWJMSCs in other experimental groups (Fig. 9). MOG was also expressed in both RECA-induced groups. Nevertheless, high protein expression of MOG was only observed when hWJMSCs were induced with a higher concentration of RECA, i.e. 1200 μg/ml.

### Effects of RECA on the cell cycle of differentiated hWJMSCs

**Fig. 10:**
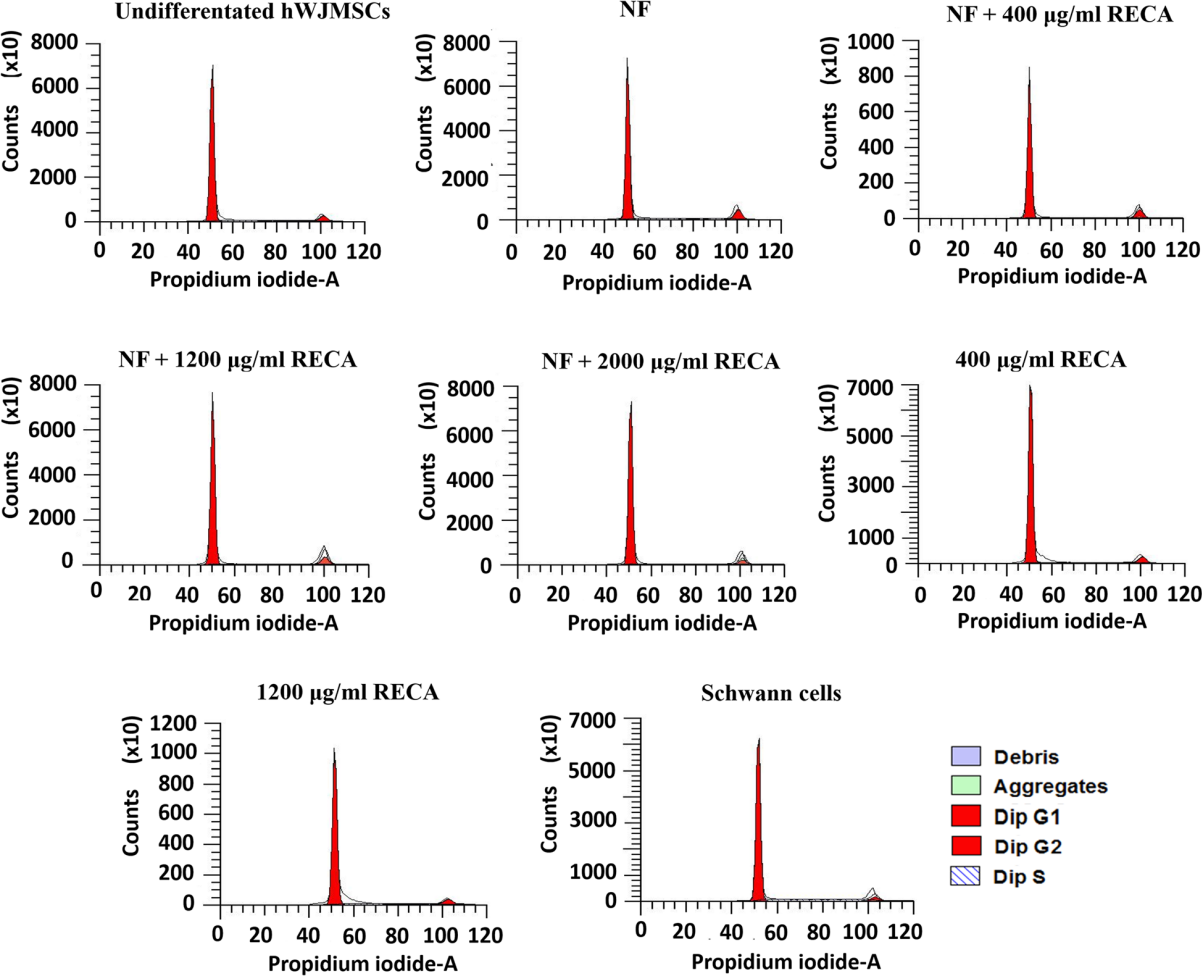
DNA histogram showing the distribution of the hWJMSC population in each experimental group for different cell cycle phases (G_0_/G_1_, S and G_2_/M phase). The x-axis represents the relative fluorescence intensity proportional to the DNA content. A similar cell cycle pattern was observed in all experimental groups, and most of the cultured cells were in the G_0_/G_1_ phase (*n* = 3)

The DNA content of hWJMSCs was assessed following the induction period to determine the cell cycle pattern. Cell cycle analysis showed that hWJMSCs induced with RECA exhibited a normal cell cycle pattern (Fig. 10). A similar pattern was also seen in undifferentiated hWJMSCs and Schwann cells.

## Discussion

*C. asiatica* (L.) is well known for its versatility for treating several illnesses and has served as a brain tonic for decades [28]. Foreseeing its potential as a nerve stimulant, its neurotrophic properties has been scrutinized and scientific investigations have shown that it has the capability of inducing the neurites extension and dendritic arborization of neurons [29, 30]. However, its stimulatory effects on the development of peripheral neural cells is still not well-documented. Therefore, the present study attempted to investigate the capacity of *C. asiatica* (L.), in place of recombinant trophic factors, to induce the differentiation of hWJMSCs into Schwann cell lineage in vitro. The use of hWJMSCs could serve as an alternative cell source for the mass production of Schwann-like cells to treat patients with peripheral nerve injuries in future.

The present study shows that the supplementation of RECA in culture reduced the viability and proliferation of hWJMSCs. RECA exerted anti-proliferative effects on hWJMSCs in a dose-dependent manner, as at higher concentrations ≥1200 μg/ml, it significantly inhibited cell proliferation. Similar trend in the inhibition of cell growth was also reported on human cornea and respiratory epithelium cells upon supplementation of *C. asiatica* (L.) extract at higher concentrations [31, 32]. The inhibitory effects of *C. asiatica* (L.) might be due to the presence of madecassoside and asiaticoside in the *C. asiatica* (L.) extract, which inhibit the proliferation of keratinocytes [33]. However, the inhibitory effects of RECA on the proliferation of hWJMSCs in this study might not be due to those compounds. This is because madecassoside and asiaticoside were present in small amounts in RECA, which differs from the extracts used in other studies that had higher amounts of the bioactive compounds in *C. asiatica* (L.) [34, 35]. The growth inhibition of hWJMSCs in the present study may be due to the effects of the high amount of phenolic compounds in RECA. At physiological levels, phenolic compounds, including flavonoids, possess antioxidant activity and can protect mammalian cells from oxidative stress. However, an overdose of flavanoids can induce a stressful environment to cells, which leads to cellular damage [36]. This is because, at higher concentrations, flavonoids have a greater tendency to act as pro-oxidants and generate oxidative stress, thought to be due to the hydroxyl group in its structure [37]. Thus, cells might be unable to neutralize oxidized flavonoids, which consequently lead to a high degree of oxidative stress and ultimately cellular injury [38].

In the present study, oxidative stress was clearly seen as the concentrations of RECA increased beyond 1200 μg/ml. In this RECA concentration range, the cell membrane and composition of hWJMSCs might have deteriorated following oxidative stress. Such impairment might cause the cells to be unable to regulate their physiology, which in turn may have caused the imbalanced movement of water and electrolytes in to and out of the cells [39]. This caused the accumulation of water inside the cells, which made the cells look distended and cellular organelles appeared to bulge out on the cell surface, as shown in the morphology of hWJMSCs treated with 1200 μg/ml RECA. A further increase in the RECA concentration up to 2400 μg/ml created a very stressful oxidative environment for the hWJMSCs, which consequently caused cell death at the end of the culture period. Nevertheless, the effects of oxidative stress seemed to be insignificant in hWJMSCs cultured in induction medium with NF + 2000 μg/ml RECA. This might be due to the presence of several neurotrophic factors such as bFGF, PDGF-AA and neuregulin in the induction medium, which serve as mitogenic inducers in cells [40–42].

Although a high concentration of RECA reduced the viability of hWJMSCs, it exhibited better neural-inducing activity. This could be due to the composition of bioactive compounds and phenolic contents in RECA. A very low composition of bioactive compounds in RECA required the supplementation of RECA to the cells at a high concentration. Unfortunately, higher RECA concentrations has simultaneously increased the composition of phenolic contents in the extract. Consequently, the hWJMSCs that were induced with RECA alone, particularly at 1200 μg/ml RECA, expressed all the neural protein markers at high levels despite experiencing growth inhibition.

The proliferative and neural inductive effects of RECA at concentrations below 400 μg/ml (3.125–200 μg/ml), either in RECA alone or in combination of NF (NF + RECA) were also tested in the preliminary work of this study. The findings demonstrated that those concentrations were anti-proliferative to hWJMSCs in dose and time-dependent manner. In the neural differentiation assay, RECA below a dose of 400 μg/ml was found to be inefficient to induce hWJMSCs to neural cell lineages. The induced hWJMSCs, either in RECA alone or RECA in combination of neurotrophic factors (NF), did not express S100β, p75 NGFR, MBP, GFAP and MOG neural protein markers (data not shown).

The protein expression of GFAP and MOG were also assessed in the present analysis. GFAP is a neural-specific marker of astrocyte, while MOG is an oligodendrocyte-specific marker [43, 44]. The use of these markers was to investigate whether RECA can stimulate the commitment of hWJMSCs into other neural lineage cells besides Schwann cell lineages. The current findings show that RECA by itself does not specifically induce hWJMSCs into the Schwann cell lineage. The expression of GFAP and MOG in hWJMSCs induced with RECA alone at 400 μg/ml and 1200 μg/ml of RECA indicated that RECA has the potential to differentiate hWJMSCs into other neural cell lineages as well. This observation highlights the importance of combining RECA with neurotrophic factors, which serve as inducers to elicit the differentiation of hWJMSCs into a specific neural lineage [45]. RECA, which is derived from a plant, may act synergistically with synthetic growth factors to facilitate the neural differentiation of hWJMSCs [46].

The present study revealed that not all of the differentiated hWJMSCs in the various NF + RECA groups displayed similar protein expression to that of Schwann cells. This was probably due to the alternate expression of neural markers that is regulated differentially at every stage of Schwann cell development [47]. Hence, the development of Schwann cells should be characterized by various Schwann cell-related markers at different time points. Additionally, Sox10 marker is also suggested for the identification of Schwann cells [48]. This is because this marker continues to be expressed in these cells throughout their development and would be of great help for distinguishing Schwann cells from other neural cell types. Several lines of evidence have demonstrated that some Schwann cell-specific markers can also be expressed by non-neural cells, such as mesenchymal stem cells, which have been reported to express S100β, similar to what was observed in undifferentiated hWJMSCs in the current study [49, 50]. The absence of MBP in NF-induced hWJMSCs suggests that the cells might have developed into non-myelinating Schwann cell-like cells. This is because only mature and myelinating Schwann cells express MBP. However, the expression of neural markers was assessed using a qualitative approach, based on the intensity of protein marker expression by fluorescent imaging in the current study. Therefore, it would be a great of value in practice if the current findings can be supported by quantitative data from either Western blotting or flow cytometry.

The contradictory findings between the gene and protein expression analysis in the present study are in parallel to those reported in other study [51]. The discrepancy in the results might have been caused by several factors. First, it could be due to the different half-life of mRNA and protein of the neural markers [52]. It is possible for mRNA to disappear rapidly, but protein persists in cells [53]. Second, interactions between microRNA (miRNA) and mRNA of the tested neural markers may have occurred. In fact, miRNA is a post-transcriptional regulator of messenger RNA (mRNA) that controls gene expression in mammalian cells [54]. It degrades mRNA upon transcription in a sequence-specific manner, which causes the transcription level of mRNA to decrease [55]. As translated proteins are not influenced by miRNA, they would accumulate in the cellular pool. Therefore, it may be possible for a cell to have a low abundance of mRNA and high expression of the corresponding protein. Finally, an inappropriate time point for analysis of the gene and protein expression could also contribute to discrepancy in the results [51]. In the present study, the analysis of gene and protein expression was performed at the end of the induction period. This kind of approach might affect the accuracy of mRNA detection in cells. In view of this limitation, the present study supports finding a critical time point for sample collection and analysis; perhaps gene and protein expression analyses should be performed at different time points during cell stimulation, so the expression of neural markers in differentiating cells can be detected precisely during induction.

The cell cycle analysis demonstrated that treatment with RECA did not change the life cycle of differentiated hWJMSCs, regardless of whether it was induced by RECA alone or RECA in combination with NF. Both the differentiated and undifferentiated hWJMSCs exhibited a normal cell cycle pattern, in contrast to tumor cells, which have a high percentage of cells in the S-phase [56]. The cell distribution showed that cells were mainly in the G_0_/G_1_ phase in the present study, which could be attributed to the confluent state of the cells following induction and/or the inhibitory effects of RECA itself [57]. Both the factors have the possibility to impede cells from continuously dividing, which consequently causes cell arrest at the quiescent stage (G_0_/G_1_ phase).

It has been known that the composition of bioactive components of *C. asiatica* (L.) varies according to its geographical distribution. The utilization of RECA, as a crude extract from local *C. asiatica* (L.) plant, in the present study has allowed the composition of bioactive components of the local *C. asiatica* (L.) plant and its efficacy in stimulating the neural differentiation of stem cells to be determined. The effectiveness of the RECA in inducing the neural differentiation of stem cells provided new insights for the industry to produce neurotrophic products from local *C. asiatica* (L.) plant. In addition, the local production of plant-based nerve stimulant could reduce the production cost, as *C. asistiaca* (L.) is readily available in Malaysia. Furthermore, the current findings of this study can serve as a guideline for improving the method of cultivation of *C. asiatica* (L.) plant, extraction and desolvation method for yielding high quality of *C. asiatica* (L.) extract in future.

Since the current findings are focused on the cell cycle pattern, the present study suggests that DNA ploidy should be estimated throughout the assay. The underlying mechanism of action of RECA regarding the proliferation and neural differentiation of hWJMSCs is also worth elucidating in further analysis. Further investigations using in vivo models are also warranted to ensure that RECA is effective in differentiating hWJMSCs into Schwann cells before translating these findings into clinical practice.

## Conclusions

In conclusion, RECA at 400 μg/ml and 1200 μg/ml as either alone or in combination of NF has the potential to induce the differentiation of hWJMSCs to Schwann cells and other neural cell lineages. However, considering the toxicity effects of RECA at higher concentration, as safer dose of RECA (400 μg/ml), which is below the IC_50_, is suggested to be effective to induce the neural differentiation of hWJMSCs. RECA at this dose had mild anti-proliferative effects on hWJMSCs, retained normal cell cycle pattern as well as stimulated a distinct expression of neural protein markers on the differentiated cells. It has a potential to be used as an alternative nerve stimulant for nerve regeneration in future.

## Additional file

Additional file 1 : **Figure S1** HPLC chromatogram of raw extract of *C. asiatica* (L.), (RECA). Reproduced with permission [58]. (DOCX 81 kb)

## Data Availability

The authors declare that the datasets supporting the conclusion of this article are included within the article and its Additional file.

## Abbreviations

ATRA: All-trans retinoic acid
GAPDH: Glyceraldehyde 3-phosphate dehydrogenase
GFAP: Glial fibrillary acidic protein
hMSCs: Human mesenchymal stem cells
hWJMSCs: Human Wharton’s jelly derived-mesenchymal stem cells
IC_50_: Half maximal inhibitory concentration
MBP: Myelin binding protein
NF: Neurotrophic factors
p75 NGFR: Low-affinity nerve growth factor receptor
PDGF-AA, bFGF: Basic fibroblast growth factor
RECA: Raw extract of *C. asiatica* (L.)
β-ME: Beta-mercapatoethanol
