# Supplementary material for: The effects of Centella asiatica (L.) Urban on neural differentiation of human mesenchymal stem cells in vitro
Source: BMC Complement Altern Med. 2019 Jul 8;19:167. doi: 10.1186/s12906-019-2581-x (PMC6615117; doi:10.1186/s12906-019-2581-x)
Supplement: Supplementary file 1 — : Figure S1 HPLC chromatogram of raw extract of C. asiatica (L.), (RECA). Reproduced with permission [58]. (DOCX 81 kb) [file 12906_2019_2581_MOESM1_ESM.docx]

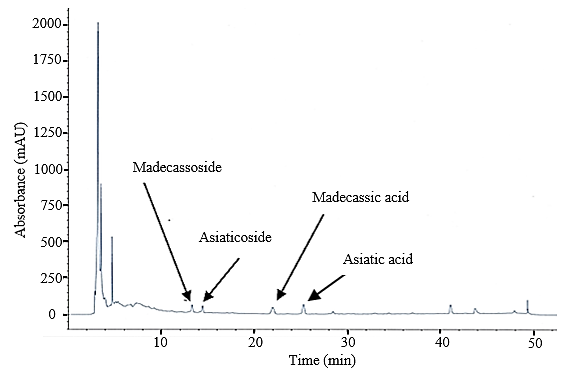


Additional file 1: Figure S1. HPLC chromatogram of raw extract of *C. asiatica* (L.), (RECA). Reproduced with permission [75].
